# Can mild cognitive impairment and Alzheimer’s disease be diagnosed by monitoring a miRNA triad in the blood?

**DOI:** 10.1111/acel.13627

**Published:** 2022-05-10

**Authors:** Zhuang‐Yao D. Wei, Ashok K. Shetty

**Affiliations:** ^1^ Institute for Regenerative Medicine Department of Molecular and Cellular Medicine Texas A&M University Health Science Center College of Medicine College Station Texas USA

**Keywords:** age‐related cognitive impairment, Alzheimer's disease, biomarkers, dementia, mild cognitive impairment, miRNAs

## Abstract

Objectively diagnosing age‐related cognitive impairment (ACI), mild cognitive impairment (MCI), and early‐stage Alzheimer's disease (AD) is a difficult task, as most cognitive impairment is clinically established via questionnaires, history, and physical examinations. A recent study has suggested that monitoring a miRNA triad, miR‐181a‐5p, miR‐146a‐5p, and miR‐148a‐3p can identify ACI and its progression to MCI and AD (Islam et al., *EMBO Mol Med*. **13**: e14997, 2021). This commentary deliberates findings from this article, such as elevated levels of the miRNA triad in the brain impairing neural plasticity and cognitive function, the efficiency of measuring the miRNA triad in the circulating blood diagnosing MCI and AD, and the promise for improving cognitive function in MCI and AD by inhibiting this miRNA triad. Additional studies required prior to employing this miRNA triad in clinical practice are also discussed.

## Graphical abstract

Promise of measuring a miRNA triad in the circulating blood as a biomarker of mild cognitive impairment (MCI) and Alzheimer's disease (AD), and the likely contribution of the miRNA triad to impaired neural plasticity and cognitive function in MCI and severe cognitive dysfunction in AD.

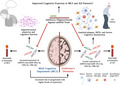

**Abbreviations**
ACI: age‐related cognitive impairment
AD: alzheimer's disease
APPPS: amyloid precursor protein/presenilin
CNS: central nervous system
IL‐1b: interleukin‐1 beta
MCI: mild cognitive impairment
miRNA: microRNA
mRNA: messenger RNA
MMSE: mini‐mental state examination
ncRNA: noncoding RNA
RISC: RNA‐induced silencing complex
TNF‐alpha: tumor necrosis factor‐alpha

Aging is coupled with a progressive decline in cognitive abilities in a significant percentage of individuals. Such age‐related cognitive impairment (ACI) is characterized by declines in the overall gray matter and hippocampus volumes (Harada et al., 2013; Sanford, 2017), with some changes commencing at ~20 years of age (Terry & Katzman, 2001). Many factors increase ACI risk, including neuroinflammation, diabetes, depression, hypothyroidism, number of surgeries, and cannabis use (Canet et al., 2003; Kodali et al., 2021; Luboshitzky et al., 1996; Shrivastava et al., 2011; Simen et al., 2011). Aging is also associated with pathological cognitive declines, including mild cognitive impairment (MCI) and Alzheimer's disease (AD). MCI is typified by a greater cognitive decline than ACI but not severe enough to hinder daily living activities (Sanford, 2017). While ACI does not typically progress to dementia, a significant percentage of individuals with MCI progress into dementia or AD within five years of MCI diagnosis (Gauthier et al., 2006; Petersen et al., 2001). MCI affects 3–19% of adults older than 65 years old and is likely caused by several factors, including cholinergic neuronal loss, cerebrovascular disease, and amyloid deposition (Gauthier et al., 2006; Mufson et al., 2000). Dementia is typified by more severe and widespread cognitive and mood deficits, substantially interfering with daily function (Gauthier et al., 2006). The overall incidence of dementia has declined due to modifiable environmental factors (Satizabal et al., 2016). However, the number of people with dementia has enlarged due to increased life expectancy resulting from advances in public health, improved management of behavioral and social risk factors, and progress against cardiovascular diseases (Olshansky, 2015; Shetty et al., 2018).

An accurate diagnosis of ACI versus MCI, MCI versus AD, or predicting the progression of MCI into AD is challenging. Currently, the diagnostic criteria are primarily clinical and done with a comprehensive history, mini‐mental state examination (MMSE), and neurological investigations. However, MMSE scores cannot objectively diagnose cognitive impairment because the results depend on patients’ attention state, cooperation, educational, and occupational background. On the contrary, laboratory and radiological studies are mostly done to rule out the other causes of dementia (Knopman et al., 2003). Specific biomarkers that could be used consistently to diagnose ACI, MCI, or AD are yet to be discovered. This commentary discusses the recent findings by Islam and colleagues that the cognitive status of individuals could be gleaned from studying changes in a microRNA (miRNA) triad in the circulating blood (Islam et al., 2021).

miRNAs are ~22 nucleotides long, have a post‐transcriptional/translational regulatory target, and have diverse sequences. miRNAs can have tissue‐specific expression patterns, and hence, changes in the characteristic of miRNAs are often used as a marker for disease (Zeng, 2006). miRNA genes are transcribed by RNA polymerase II to create a primary miRNA, which is processed first in the nucleus to form a 70‐nucleotide long hairpin precursor and then cleaved by dicer to form the mature 22 nucleotide miRNA (Du & Zamore, 2005; Bushati & Cohen, 2007). A dicer protein complex also forms the RNA‐induced silencing complex (RISC), which incorporates the mature miRNA to block, cleave or degrade the target mRNA (Bushati & Cohen, 2007).

Aging can influence the miRNA expression, with at least 115 miRNAs showing an association with aging (Somel et al., 2010). In mice, 70 different miRNAs display upregulation during brain aging, with 27 of those targeting genes of mitochondrial complexes involved in oxidative phosphorylation (Li et al., 2011). Changes in miRNA expression have also been considered to track the development of MCI. For example, the miR‐132 family can distinguish MCI from ACI with 84–94% sensitivity and 96–98% specificity (Sheinerman et al., 2013). Furthermore, in AD, four miRNAs, miR‐31, miR‐93, miR‐143, and miR‐146a, are downregulated in the serum (Dong et al., 2015). Therefore, decreased or increased levels of specific miRNAs could be used as a biomarker of MCI or AD.

In an elegant study, Islam and colleagues discovered changes in a miRNA triad in the circulating blood that can assist in tracking the development of cognitive impairment and identify CNS pathological states that can progress into a declined cognitive state (Islam et al., 2021). In a mouse model, the study found a link between age‐related spatial reference memory impairment and 55 differentially expressed miRNAs in the circulating blood. Notably, three miRNAs, miR‐181a‐5p, miR‐146a‐5p, and miR‐148a‐3p, capable of impacting decisive processes in preserving cognitive function in healthy people (Marioni et al., 2018), were substantially elevated. Since the principal biological activities affected by the expected targets of miR‐181a‐5p, miR‐146a‐5p, and miR‐148a‐3p comprise neuronal plasticity, GTPase‐mediated signal transduction, and the response to transforming growth factor‐beta, among others, the results suggested that these three miRNAs likely control vital processes linked to cognition that are dysregulated in ACI. Further analysis revealed that this miRNA triad also undergoes significant upregulation in the mouse brain from 13.5 to 16 months, coinciding with the learning impairment seen at 16.5 months. Such results implied that the increased expression of the miRNA triad preceded age‐related cognitive decline, and increased blood levels of the three microRNAs denote pathophysiology in the brain (Islam et al., 2021).

Cell culture analysis revealed that miR‐181a‐5p and miR‐148‐3p were highly expressed in neurons. Administration of miR‐181a‐5p and miR‐148‐3p mimics led to downregulation of genes linked to synaptic plasticity and learning and memory, consistent with the role of these miRNAs in neurodegenerative diseases (Chen et al., 2019; Stepniak et al., 2015). In contrast, miR‐146a‐5p was highly enriched in microglia, and increased levels of miR‐146a‐5p in microglia cultures resulted in the downregulation of genes linked to ncRNA processing and protein folding, the upregulation of proinflammatory cytokines, interleukin‐1 beta (IL‐1b), IL‐6, and tumor necrosis factor‐alpha (TNF‐alpha) and downregulation of the antiinflammatory cytokine IL‐10, consistent with the role of miR‐146a‐5p in inflammatory processes (Maschmeyer et al., 2018). Next, the authors compared gene expression changes mediated by the miRNA triad overexpression with the gene expression observed in CK‐P25 mice, a mouse model for AD‐like neurodegeneration (Fischer et al., 2005) and human AD patients. The results suggested that the genes and proteins downregulated in AD patients strongly overlapped with the downregulated genes observed in response to miR‐148a‐3p and miR‐181a‐5p. Furthermore, the addition of mimic oligonucleotides representing the 3‐miRNAs to mouse primary hippocampal cell cultures reduced the number of synapses and aberrant neuronal activity (Islam et al., 2021).

The above results collectively suggested that elevated levels of miR‐181a‐5p, miR‐146a‐5p, and miR‐148a‐3p are detrimental to neural plasticity and cognitive function. Islam and associates next explored whether the miRNA triad in the circulating blood could detect MCI and AD (Islam et al., 2021). Indeed, the miRNA triad was significantly elevated in MCI patients compared to healthy patients, implying that elevated levels of these miRNAs signify increased cognitive impairment. Moreover, the study revealed a higher expression of miRNA triad in MCI patients advancing to AD, highlighting that the miRNA triad is elevated in MCI but undergoes further upregulation in MCI patients at risk for developing AD. Thus, the detection of the miRNA triad in the circulating blood could serve as a specific molecular marker to infer the cognitive status of patients. Furthermore, injection of a combination of inhibitory oligonucleotides against the miRNA triad into the hippocampus of 16.5‐month‐old mice resulted in the downregulation of the three miRNAs and better hippocampus‐dependent memory function. When the inhibitory oligonucleotides against the miRNA triad were injected into the hippocampus of 7‐month‐old amyloid precursor protein/presenilin (APPPS) transgenic mice, a model of AD, the three miRNAs were downregulated, and hippocampus‐dependent learning and memory functions were improved. Thus, the miRNA triad could be used not only as a biomarker for detecting cognitive decline but also as a potential therapeutic target (Islam et al., 2021).

Currently, when a patient sees a doctor for age‐related cognitive problems, there are hardly any objective tests that could be used to precisely diagnose ACI, MCI, or AD. Typically, the physician takes a history, performs routine examination, and conducts cognitive function tests that are available at their disposal to assess the possibility of ACI, MCI, AD, or any other conditions causing cognitive impairment. Laboratory and radiological studies are only being utilized to determine causes such as vitamin B12 deficiency or hypothyroidism. There are no specific tools to track the extent of cognitive decline or differentiate ACI, MCI, and AD. Therefore, the finding by Islam and colleagues that a triad of specific miRNAs is upregulated in MCI and increases further in MCI patients progressing into AD has implications (Islam et al., 2021). The study also suggested that the miRNA triad could be a therapeutic target. Inhibition of the miRNA triad expression led to a better cognitive function in both aged and AD mice. Thus, investigation of this triad of miRNAs in the blood can serve as a specific biomarker to screen patients at risk of developing pathological cognitive impairment and track patients who already have MCI and are at risk of developing AD. Such screening also allows the application of promising therapeutic interventions to at‐risk patients or to halt the progression of cognitive decline in patients diagnosed with MCI or early‐stage AD.

In summary, Islam and colleagues discovered a miRNA triad that promises to serve as a biomarker for ACI, MCI, and AD and a potential target for improving cognitive function in MCI and AD patients (Islam et al., 2021). A cartoon summarizing the utility of evaluating the miRNA triad in the circulating blood as a biomarker of MCI and AD, and the miRNA triad serving as one of the mechanisms underlying impaired neural plasticity and cognitive function in MCI and severe cognitive dysfunction in AD is illustrated (Figure 1). Although these findings are exciting, additional studies are needed before routinely employing this miRNA triad in clinical practice. The specificity of the triad of miRNAs for diagnosing MCI and AD need to be further validated with larger sample sizes. Furthermore, there is a concern that the three miRNAs suggested as biomarkers of MCI/AD are also implicated in several other pathological processes. For example, miR‐181a‐5p has been associated with colorectal cancer (Han et al., 2017), osteoarthritis (Xue et al., 2018), and obesity (Lozano‐Bartolomé et al., 2018). Significantly, all three miRNAs are associated with gastric cancer (Chen et al., 2013; Li et al., 2014; Song et al., 2018).

**FIGURE 1:**
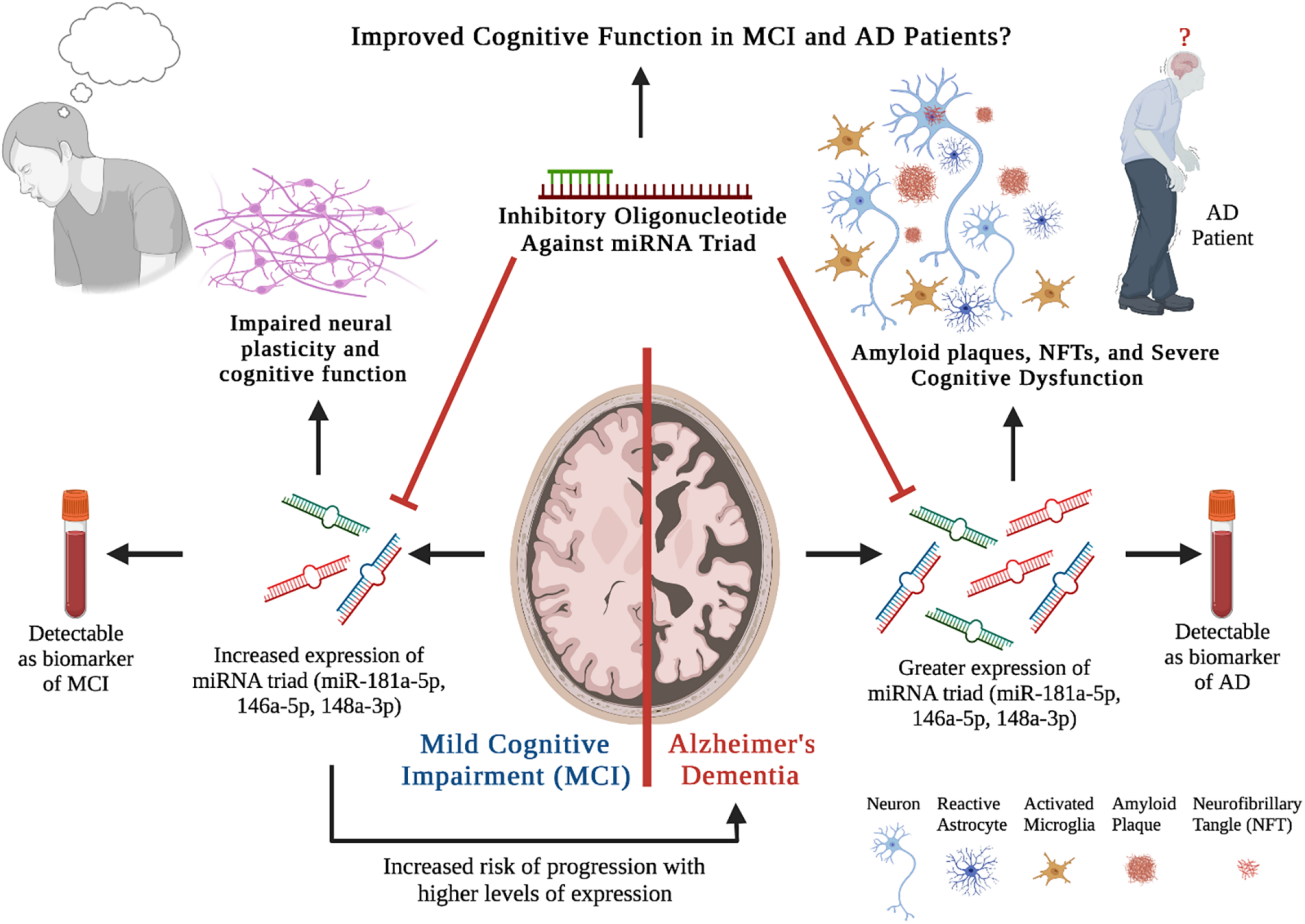
The cartoon shows the promise of measuring miRNA triad (miRs, 181a‐59, 146a‐5p, and 148a‐3p) in the circulating blood as a biomarker of mild cognitive impairment (MCI) and Alzheimer's disease (AD), and the miRNA triad serving as one of the mechanisms underlying impaired neural plasticity and cognitive function in MCI and severe cognitive dysfunction in AD. Increased risk of MCI progression into AD with higher levels of miRNA triad expression and the promise of an inhibitory oligonucleotide against the miRNA triad in improving cognitive function in MCI and AD are also indicated

The above issues raise the possibility that an underlying unrelated medical issue elevating the miRNA triad could lead to a false‐positive MCI/AD diagnosis, causing a devastating effect on the patient. Therefore, steps need to be implemented to evaluate the extent of misdiagnosis frequency and probability before being widely recommended in clinical practice. A standardized extent of miRNA triad elevation needs to be established in MCI/AD patients lacking other diseases vis‐à‐vis MCI/AD patients with gastric cancer or patients with only gastric cancer. Moreover, the extent of miRNA triad elevation in aged and AD animal models with or without gastric cancer or other disease states might reveal statistically significant differences in miRNA triad levels in MCI/AD versus MCI/AD plus other diseases (Benedetti et al., 2021; Carella et al., 2021; Moreira‐Costa et al., 2021). Also, when patients present with other significant medical issues along with symptoms of MCI/AD, the degree of miRNA triad elevation would need to be assessed and compared to a standard set of values to discern whether the range falls within MCI/AD only, MCI/AD plus a concomitant medical condition, or only a confounding medical condition. However, such interpretation assumes that the degree of miRNA triad elevation is directly linked to the number of disease conditions a patient has. Alternatively, the measurement of the miRNA triad in autopsied brain samples from patients who have passed away from disease conditions unrelated to MCI/AD may provide the extent of the false‐positive rate if a certain percentage of patients exhibit elevated miRNA triad levels in the absence of any behavioral and pathological hallmarks of MCI/AD. Calculations can also be done for false negative, true negative, and true‐positive rates with a similar study design. After such statistical studies provide a standardized value of miRNA triad elevation in different conditions, clinicians can better interpret miRNA triad results and explain them to the patient.

Finally, regarding the use of miRNA triad as a potential target for improving cognitive function in MCI and AD patients, additional studies investigating the effects of administration of miRNA triad inhibitors on other organ systems are needed. For example, miR‐181a‐5p has been shown to inhibit cancer cell migration and prevent cancer metastasis. Therefore, global inhibition of this miRNA to treat MCI or AD may be detrimental, exposing the patient to oncogenic effects (Li et al., 2015). Transient brain‐specific inhibition of the miRNA triad is likely an alternative to avoid adverse systemic effects, which needs the development of advanced noninvasive approaches to accomplish that. Nonetheless, the miRNA triad is an exciting new development that promises to serve as a biomarker for ACI, MCI, and AD, with relevant additional investigations.

## AUTHOR CONTRIBUTIONS

Zhuang‐Yao Daniel Wei (Z‐YDW) prepared the first draft of the manuscript text and the figure. Ashok K. Shetty (AKS) provided feedback to the first draft, edited, and finalized the manuscript text and the figure.

## CONFLICT OF INTEREST

The authors declare that there are no competing interests.

## Data Availability

All data needed to evaluate the conclusions of this commentary are present in the paper.
